# Putative Novel Genotype of Avian Hepatitis E Virus, Hungary, 2010

**DOI:** 10.3201/eid1808.111669

**Published:** 2012-08

**Authors:** Krisztián Bányai, Ádám György Tóth, Éva Ivanics, Róbert Glávits, Katalin Szentpáli-Gavallér, Ádám Dán

**Affiliations:** Hungarian Academy of Sciences, Budapest, Hungary (K. Bányai);; and Veterinary Diagnostic Directorate, Budapest (Á.G. Tóth, É. Ivanics, R. Glávits, K. Szentpáli-Gavallér, Á. Dán)

**Keywords:** chickens, avian hepatitis E virus, aHEV, big liver and spleen disease, hepatosplenomegaly, viruses, Hungary, hepatitis E virus, genotype, genetic diversity

## Abstract

To explore the genetic diversity of avian hepatitis E virus strains, we characterized the near-complete genome of a strain detected in 2010 in Hungary, uncovering moderate genome sequence similarity with reference strains. Public health implications related to consumption of eggs or meat contaminated by avian hepatitis E virus, or to poultry handling, require thorough investigation.

Avian hepatitis E virus (aHEV) is a causative agent of big liver and spleen disease (BLSD) in chickens (*1*), which manifests itself, as its name indicates, by hepatosplenomegaly. The etiologic association has been demonstrated worldwide, including Australia, North America, Asia, and Europe (*2**,**3*). In Hungary, BLSD was observed during the 1980s, but association of the disease with hepatosplenomegaly was not made in Hungary until 2008 (*4**,**5*). Ivanics et al. (*5*) reported outbreaks of aHEV infection and increased deaths of chickens caused by hepatitis and splenitis and reduced egg production in chicken flocks in Hungary. Tissue samples from chickens collected during 2010 were positive for aHEV by reverse transcription PCR (RT-PCR) (*6**,**7*). Paired samples indicated seroconversion upon inoculation as measured by serologic assay, strongly suggesting an etiologic association between BLSD and aHEV infection in the affected chicken flocks (*5*).

The aHEV is characterized by a small, nonenveloped virion and a 6.6-kb, capped, poly-A tailed single-stranded RNA genome. The genome encodes 3 open reading frames (ORFs). ORF1 is a polyprotein encoding putative functional domains of methyltransferase, papain-like cystein protease, helicase, and RNA polymerase. The capsid protein is encoded by ORF2; ORF3 encodes a multifunctional cytoskeleton-associated protein linked to viral morphogenesis and pathogenesis (*1*).

aHEV is ≈50% similar at the genomic level to mammalian HEV genotypes; recent proposals classify aHEV into a different taxon, possibly a new genus, within *Hepeviridae*. GenBank now includes 5 full or nearly complete aHEV genome sequences that could be classified into 1 of the 3 recognized aHEV genotypes. These 5 prototype strains with full or nearly complete genome sequences include a genotype 1 strain from Australia, 2 genotype 2 strains from the United States, and 2 genotype 3 strains from Europe and the People’s Republic of China (*6**,**8**–**10*). Additional putative genotypes have been identified through analysis of an ≈130-nt fragment of the helicase domain, suggesting that diversity within aHEV is higher than recognized (*11*). Our primary objective was the genetic characterization of an aHEV strain from the 2010 outbreak in Hungary to increase understanding of the origin and evolution of these emerging pathogens.

## The Study

Preliminary sequence analysis of the amplicons obtained by the diagnostic HEV RT-PCR from the 2010 BLSD outbreaks in Hungary (strains identified by HUN prefix) showed a moderate genetic relationship with reference aHEV sequences deposited in GenBank (*5*). One strain, HUN-16773–2010, found in a sample of liver tissue, was selected for further analysis. Viral RNA was extracted by using the SV96 total RNA nucleic acid extraction kit (Promega, Madison, WI, USA). Oligonucleotides were designed for our primer walking sequencing strategy on the basis of aHEV genome sequence data (Table). Genomic fragments were amplified by using a OneStep RT-PCR Kit (QIAGEN, Hilden, Germany) as described by the manufacturer. Amplicons were subsequently subjected to direct sequencing (Biomi Ltd., Gödöllő, Hungary).

**Table:** Primers used in the generation of nearly complete genome of the novel Hungarian avian hepatitis E virus strain*

Primer name	Sequence, 5′ → 3′	Location	Orientation
F1	CAG ACT ACC ATT ATC GCC AC	2926–2945	+
R1	AGC TGG AGC TTG ACA ACT TC	3319–3338	–
R1b	AGC TGG AGC TTG ACA ACC TC	3319–3338	–
F2	GGA TGC CTG ACT GGA TGG T	3926–3944	+
F2b	CTG ACT GGA TGG TGG CTC T	3932–3950	+
R2	TTA CCA GGC GAC AGA TTC C	5395–5413	–
F3	ACT CGT GTT AAG GTA ACG GC	5430–5449	+
F3b	GTG GCT GAA GAT GGC GAG AT	5541–5560	+
F3c	GGT GTG GCT GAA GAT GGC GA	5538–5557	+
R3	CCG AGA TGG GAG GAT TTC ATA A	6629–6650†	–
F4	GCA GTT TGC AGA GTC CAA GG	36–55†	+
R4	CTG ATC AAA AGT GCT ACC CT	2909–2928	–
R4b	TGA TTG ACC CGG GGT TCG	2876–2893	–
F5	GGT GTT GAG GTC GGC ATG GT	4432–4451	+
F5b	ATA TCC AGC GCA TGC AGG	4325–4342	+
R5	ATC TAC ATC TGG TAC CGT GC	4828–4847	–
R5b	GAC GTT GTG CCA TCC TGA AG	4965–4984	–
F6	TCC TGT TCT CAC CAG TGT	400–417	+
R6	AGC CCA TAA TTA ACC ATG TC	2302–2321	–
R6b	GAT GTC GAT TTA CCG CTG CC	2413–2432	–
F7	GGT GTG GCA GAG GTT AAT GAT G	897–918	+
F7b	GAT GAT GCC TTC TGC TGC TC	937–956	+
R7	AAG ACA GCA AGG ACC TCC TC	1795–1814	–
R7b	AGG CTG ATG ATC ACG GTT GG	1883–1902	–

*The annealing temperature was set at 53°C for each primer combination. +, sense; –, antisense. †5′ and 3′ end primers were designed on the basis of sequence AM943646.

The final assembly was 6,543 bp (GenBank accession no. JN997392), with negligible amounts of missing information at the 5′ (75 nt) and 3′ ends preceding the poly A tail (36 nt). Nucleotide and deduced amino acid sequences of HUN-16773–2010 aligned well with the other 5 prototype aHEV strains. The nearly complete coding sequence (1,515 aa) with a missing 17-aa region was determined for ORF1. Predicted functional domains (*12*) were identified. The sequence similarities (Megalign; DNASTAR Inc., Madison, WI, USA) of ORF1 in comparison with genotypes 1–3 aHEV strains were 81.0%–83.9% at the nucleotide level and 93.0%–95.4% at the amino acid level.

Unique amino acid changes in the ORF1 of HUN-16773–2010 were found in 16 positions compared with the European prototype aHEV strain (not shown) (*6*). ORF2 encodes the 606-aa capsid protein. Similarities to prototype aHEV strains were 83.9%–87.3% at the nucleotide level and 98.2%–99.3% at the amino acid level. We observed 2 unique amino acid substitutions in positions A53S and Q/M473T, the second residue located within antigenic region II (*6*). ORF3 encoded an 87-aa polypeptide. This region also shared high (>92%) nt and (>90%) aa similarities with genotype 1–3 aHEVs.

**Figure A1:**
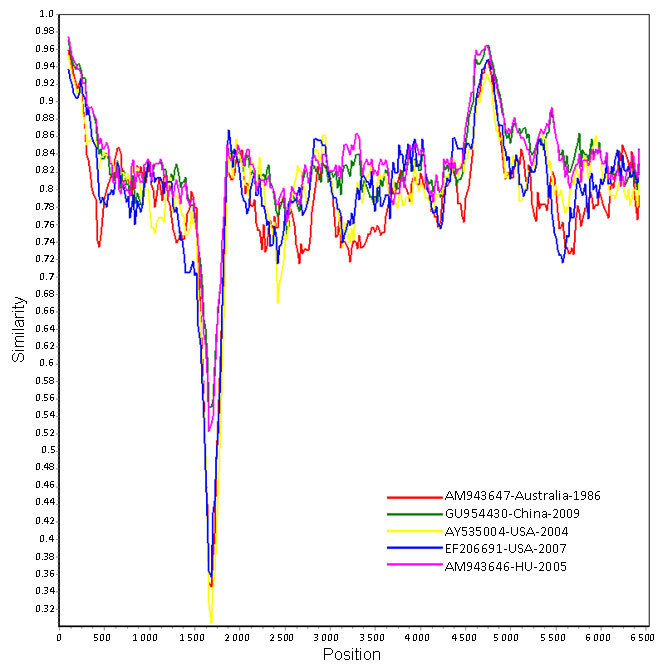
Genomewide similarity of HUN-16773-2010 to 5 other avian hepatitis E virus (aHEV) strains with nearly complete genome sequences, 1 each belonging to genotypes 1–3. Sequences were analyzed with SimPlot software (http://sray.med.som.jhmi.edu/SCRoftware/simplot) by using 200-bp window size and 20-bp step size.

When analyzing the nearly complete genome sequences, we found that strain HUN-16773–2010 shared moderate similarity to genotypes 1–3 aHEV strains (nt, 81.9%–84.9%). Similar ranges of genome sequence similarity (nt, 81.8%–82.7%) distinguish prototype aHEV strains from each other. Using SimPlot analysis (http://sray.med.som.jhmi.edu/SCRoftware/simplot), we observed this moderate nucleotide similarity of HUN-16773–2010 to the 5 prototype strains along the entire genome (Figure A1), except the near-central region of ORF1, which showed less similarity among aHEV strains; this region encodes the hinge region flanking the papain-like protease and the X domain. Similarity in the genome was highest in the 5′ end of the genome and the overlapping region of ORF2 and ORF3. We found no evidence for recombination between HUN-16773–2010 and genotypes 1–3 strains (*13*), demonstrating that this strain was not generated through recombination from prototype aHEVs.

Maximum-likelihood and neighbor-joining trees were constructed with the substitution model of Tamura and Nei, including gamma distribution shape (G) parameter (TN93+G) as implemented in MEGA 5 (*14*); 500 bootstrap replicates were used to test the reliability of the tree topology. Phylogenetic analysis of the nearly complete aHEV genome revealed 4 major clusters, where genotypes 1–3 strains formed previously recognized branches (*10*); the aHEV strain, HUN-16773–2010, appeared to form a fourth cluster (Figure, panel A). To determine whether HUN-16773–2010 shared genetic relatedness in the partial helicase gene with recently identified variants of European aHEV strains (*11*), we reanalyzed this region by using a larger sample set. Our data reaffirmed that HUN-16773–2010 forms an independent branch (not shown).

To identify the evolutionary driving forces beyond the heterogeneity of aHEV genomes, we individually ran the alignments of all 3 ORFs on the DataMonkey server by using the random effects likelihood method (www.datamonkey.org). Evidence for strong selection pressure was found at the hinge region of ORF1 involving 17 sites between amino acid positions 556 and 605 (Figure, panel B). However, 905, 384, and 3 sites within ORF1, ORF2, and ORF3, respectively, were under purifying selection across aHEV genotypes.

## Conclusions

Various genotypes of aHEV have been proposed to represent clusters of strains typical for particular geographic locations. However, new evidence has shown that some genotypes could be present on different continents (*6**,**10**,**11*). For example, although Australia seemingly remained the only continent with genotype 1 aHEV, different genotypes could be endemic to the Americas and particularly Europe, and these areas could show strain diversification. The origin of the novel aHEV strain in Hungary remains enigmatic; understanding its recent emergence awaits analysis of further isolates within and outside Europe.

Purifying selection was a key mechanism in the evolution of aHEV, suggesting the existence of a strong structural and functional constraint against diversification that would lead to extensive amino acid changes within the 3 ORFs in aHEV strains. In contrast, positive selection was predicted primarily in the hypervariable hinge region flanking the protease and X domains in ORF1. The biological role of this observation is not clear. However, recent findings have demonstrated that this region might be responsible for differences in replication efficiency (*15*). Thus, the positive evolutionary selection in this region could directly affect viral fitness and influence the pathogenesis of aHEV infections.

We provided further evidence for the marked sequence diversity among aHEVs by providing the nearly complete genome sequence of a strain identified during a BLSD outbreak in Hungary. Additional aHEV genomes could facilitate more penetrating insights into their epidemiologic features and evolutionary mechanisms and also could serve as molecular bases for reliable and robust demarcation criteria in future classification proposals.

## Figures and Tables

**Figure:**
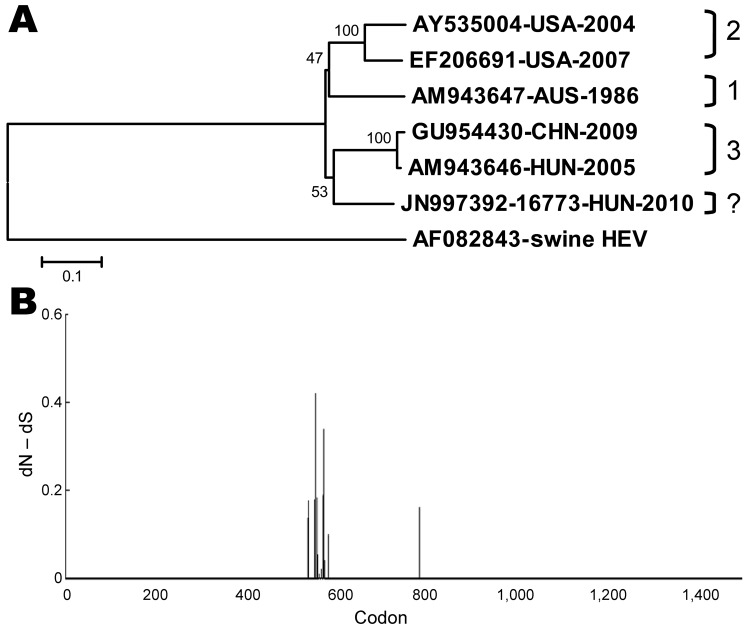
Genomic structure of HUN-16773-2010 and 5 other avian hepatitis E virus (aHEV) strains. A) Neighbor-joining tree constructed from the nucleotide sequences (*14*) of the nearly complete genome of 6 aHEV strains. Bootstrap values are indicated at branch nodes. A swine HEV served as outgroup in the phylogeny. Genotype assignments are depicted for reference aHEV genomes. Scale bar indicates genetic distance. B) Adaptive evolution of aHEV genomes. Only sites under positive selection in open reading frame 1 are shown with normalized value of dN – dS along the codon positions, as indicated by the random effects likelihood method and by using the default significance level (www.datamonkey.org).
